# Spontaneous Resolution of Recurrent Pancreatitis After Splenic Artery Pseudoaneurysm Stent Placement

**DOI:** 10.7759/cureus.50873

**Published:** 2023-12-20

**Authors:** Forrest Bohler, Giuliano Romano, Paul Eikens, David Bohler

**Affiliations:** 1 Foundational Medical Studies, Oakland University William Beaumont School of Medicine, Auburn Hills, USA; 2 Department of Surgery, Inland Imaging Associates, Missoula, USA; 3 Department of Surgery, Bitterroot Health, Hamilton, USA

**Keywords:** recurrent acute pancreatitis, portal vein thrombosis (pvt), acute pancreatitis (ap), acute pancreatitis complications, splenic artery pseudoaneurysm

## Abstract

Acute pancreatitis (AP) is a relatively common condition most often secondary to excess alcohol consumption, choledocholithiasis, medications, or hypertriglyceridemia. In rare cases, AP can result in a secondary splenic artery pseudoaneurysm (SAP). SAPs are a rare yet serious medical complication and are often under-diagnosed as they are usually asymptomatic. However, rupture and subsequent hemorrhage of SAPs pose life-threatening risks. This case involves a 72-year-old male presenting with portal vein thrombosis and recurrent episodes of AP with persistently elevated levels of lipase of no apparent etiology over a 6-month period. As patient history and pertinent test results ruled out all common causes of recurrent AP, the etiology of his AP remained unknown. After an SAP rupture and emergency treatment with an endovascular stent, the patient’s recurrent AP spontaneously resolved, and lipase returned to normal levels. This case represents a yet-to-be-reported etiology of AP in which the proximal nature of the SAP with its associated inflammatory response to the pancreas resulted in intermittent AP. The lack of any other reasonable explanation for the etiology of the patient’s recurrent AP along with the absence of any additional episodes after the treatment of his SAP supports this diagnosis. The findings of this case could prove useful to clinicians with patients suffering from recurrent episodes of AP with no known etiology and suggest that a potential undiagnosed SAP should be investigated further.

## Introduction

Splenic artery pseudoaneurysm (SAP) is a contained vascular wall lesion of the splenic artery. It is a rare yet serious condition that is underdiagnosed, as most patients are asymptomatic with the condition, and the most common complication is rupture and subsequent hemorrhage [1]. SAPs most commonly occur secondary to acute pancreatitis (AP) but also can be caused by abdominal trauma, peptic ulcer disease, and postoperative complications [2]. While they are often difficult to diagnose, cross-sectional imaging is key for diagnosis, and embolization is the first-line treatment for SAPs [3,4]. If left untreated, a ruptured SAP has a mortality rate of around 90%. [5]

Acute pancreatitis involves inflammation of the pancreas and is associated with alcohol abuse, hypertriglyceridemia, hypercalcemia, gallstones, autoimmune pancreatitis, and medication side effects. [6,7] Patients commonly present with epigastric abdominal pain accompanied by nausea and weight loss. Complications of acute pancreatitis vary based on severity, ranging from no local complications to organ failure and necrosis. [7] Other known complications of AP include portal vein thrombosis and, in rare instances, SAPs. [2,8] Fluid, nutrition, and pain management are key in the treatment of AP, as well as addressing the underlying etiology. [7]

Traditionally SAPs have been associated with AP as a rare, secondary complication, arising when digestive enzymes from the pancreas erode and weaken the vessel walls. [1] Here, we discuss a unique case in which the cause of a patient’s recurrent acute pancreatitis episodes appeared to be secondary to a splenic artery pseudoaneurysm.

## Case presentation

**Figure 1:**
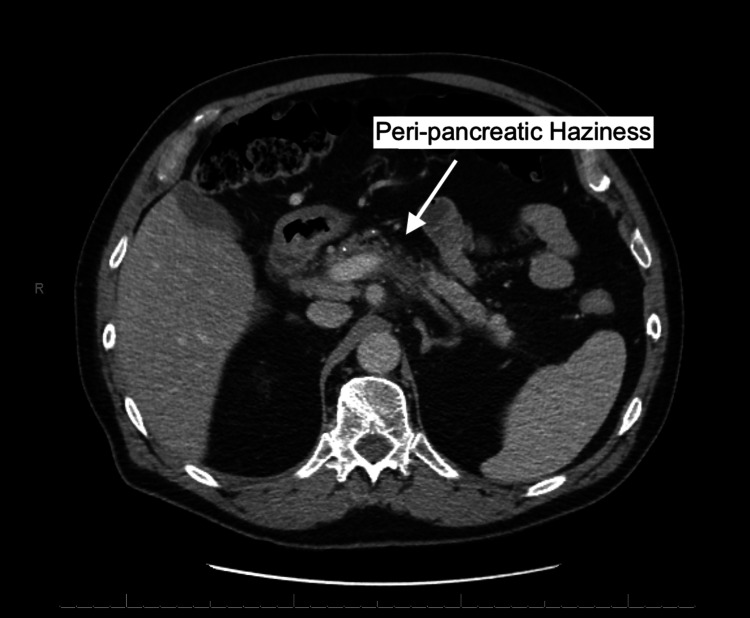
Computerized tomography scan with contrast showing peri-pancreatic haziness

**Figure 2:**
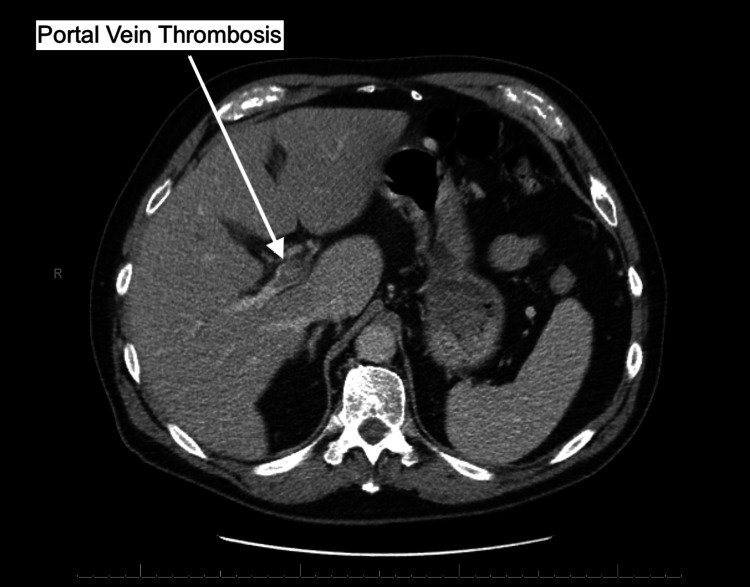
Computerized tomography scan with contrast showing portal vein thrombosis involving the majority of the intra-hepatic portal venous branches with relative sparing of the right branches

A 72-year-old caucasian male was admitted to the emergency department (ED) presenting with mid-epigastric pain that began 24 hours prior. A computerized tomography (CT) scan with contrast of the patient's abdomen revealed subtle peri-pancreatic haziness (Figure 1) with extensive portal vein thrombosis (PVT) involving the majority of the intra-hepatic portal venous branches with relative sparing of some of the right branches as seen in Figure 2.

Lab reports, as shown in Table 1, indicate elevated levels of lipase of 512 unit/L, normal triglyceride (98 mg/dL), and normal calcium levels (9.8 mg/dL). A liver function test (LFT) revealed elevated levels of total bilirubin of 2.0 mg/dL, aspartate aminotransferase (AST) of 114 unit/L, alanine transaminase (ALT) of 108 unit/L, and normal alkaline phosphatase of 81 unit/L. To rule out potential autoimmune pancreatitis, IgG4 levels were measured and found to be normal (36 mg/dL). A working diagnosis of AP with secondary PVT was made. The patient denied alcohol/recreational drug abuse, any family history of pancreatitis, recent abdominal trauma, or any previous episodes of AP. Additionally, the patient was not taking any medications known to significantly cause pancreatitis. After consultation with a gastroenterologist, the patient was made nil per os (NPO) and a 6-month anticoagulant regimen was started to treat his PVT. The patient was subsequently discharged from the ED and scheduled for a follow-up visit to monitor the progress of his anticoagulant treatment and further investigate the etiology of his AP.

**Figure 3:**
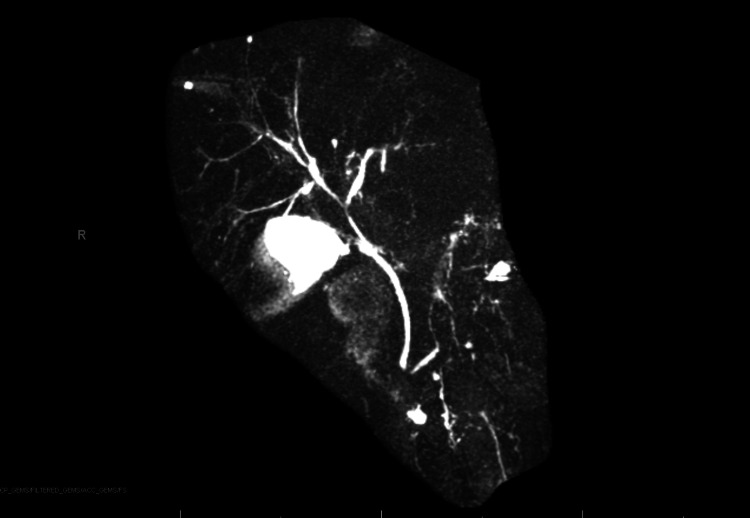
Magnetic resonance cholangiopancreatography revealing mild atrophy of the distal pancreas

An esophagogastroduodenoscopy (EGD) was performed to ensure the absence of esophageal varices and to rule out mucosal etiologies of elevated lipase, resulting in unremarkable findings. Two weeks after his EGD, magnetic resonance cholangiopancreatography (MRCP) was conducted to evaluate for any structural abnormalities of the pancreas and biliary tree, revealing normal gallbladder, cystic duct, common bile duct, and pancreatic duct. Mild atrophy of his distal pancreas was noted along with residual PVT as seen in Figure 3.

During the subsequent 4-week follow-up visit, the patient reported that he continued to experience episodes of mild intermittent epigastric pain over the previous month. Lab work revealed decreased but continually elevated levels of AST (69 unit/L) and ALT (65 unit/L) that were not unexpected due to his residual PVT along with normal levels of total bilirubin (0.9 mg/dL) and alkaline phosphatase (75 unit/L). Of particular concern, however, was that his lipase levels were increased compared to his initial ED visit at 765 unit/L. Although initial IgG4 levels were unremarkable, autoimmune-induced pancreatitis was still on the differential as not all autoimmune reactions present with elevated IgG4. A glucocorticoid trial (prednisone 40mg daily) to treat potential autoimmune pancreatitis with a 2-week follow-up was prescribed. Subsequent bloodwork 10 weeks after his initial presentation to the ED revealed normal LFT and IgG4 (24 mg/dL) but increasingly elevated lipase levels of 1,021 units/L (Table 1). Due to his continually elevated lipase levels, the patient was referred for an endoscopic ultrasound (EUS) and pancreatic biopsy. Unfortunately, since this referral was out-of-state, the patient was unable to receive an EUS until approximately 2.5 months after his referral. In follow-up visits during this period, the patient continued to experience intermittent epigastric pain, reduced appetite, and elevated lipase levels with a normal LFT. 

**Table 1:** Patient serum lab values (H) = High/Elevated N/A = Not Available

Lab Values	Initial ED Presentation	Four-Week Followup	Ten-Week Followup
Calcium (mg/dL)	9.8	9.8	N/A
Total Bilirubin (mg/dL)	2.0 (H)	0.9	1.1
Alkaline Phosphatase (unit/L)	81	75	70
Aspartate Aminotransferase (AST) (unit/L)	114 (H)	69 (H)	41
Alanine Transaminase (ALT) (unit/L)	108 (H)	65 (H)	43
Lipase (unit/L)	512 (H)	765 (H)	1,021 (H)
IgG4 (mg/dL)	36	N/A	24

**Figure 4:**
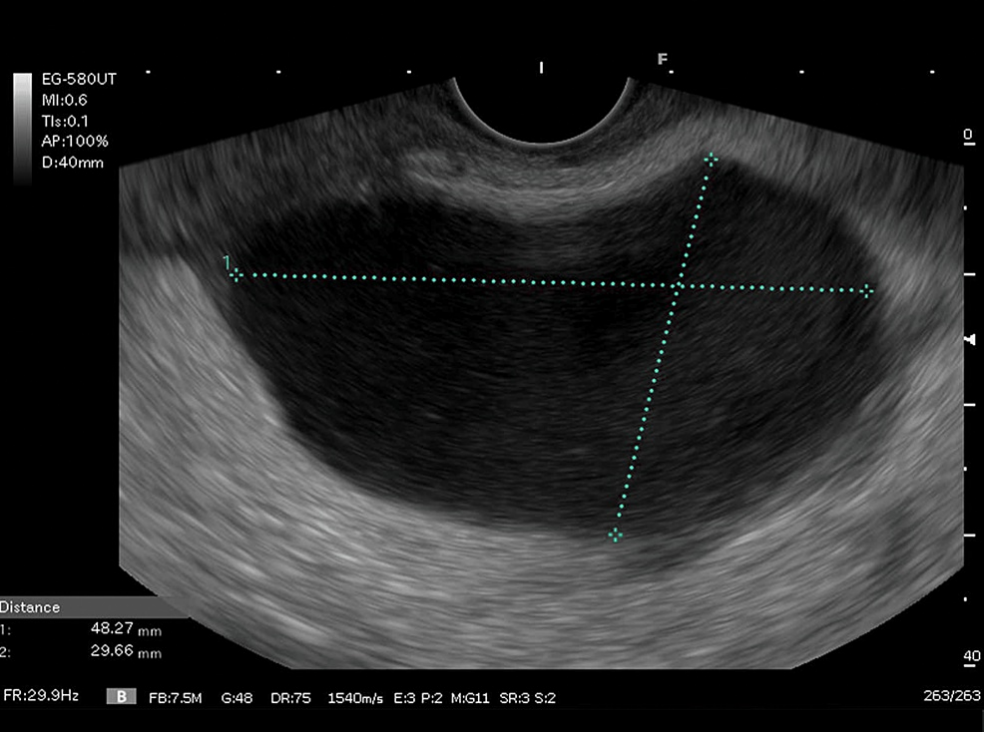
Endoscopic ultrasound showing a 48X30 mm collection that was initially suspected to be a pancreatic pseudocyst but later revealed to be a splenic artery pseudoaneurysm

**Figure 5:**
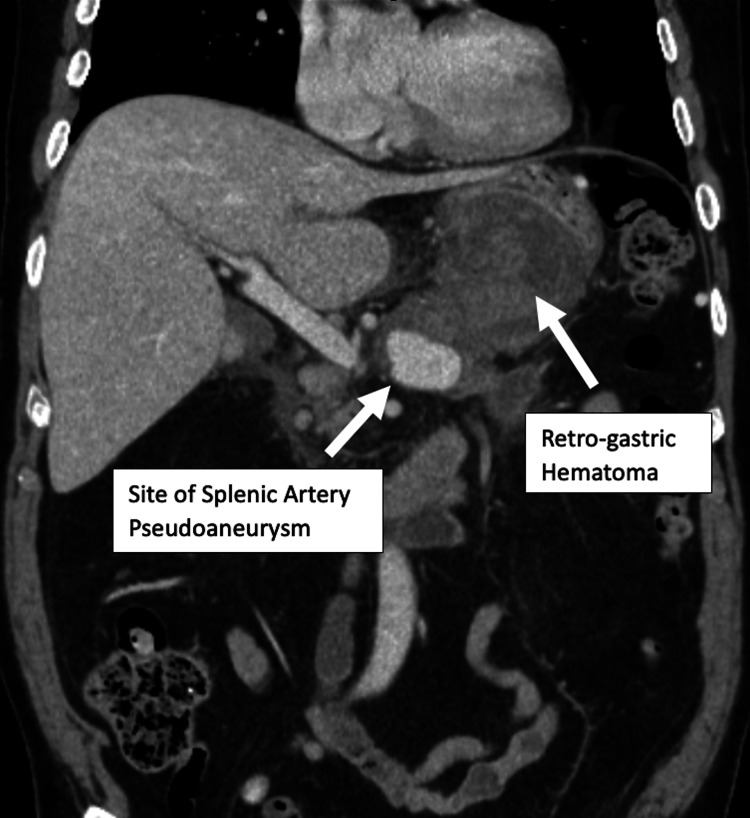
Computerized tomography scan with contrast showing the site of the splenic artery pseudoaneurysm and retro-gastric hematoma within the abdominal cavity

**Figure 6:**
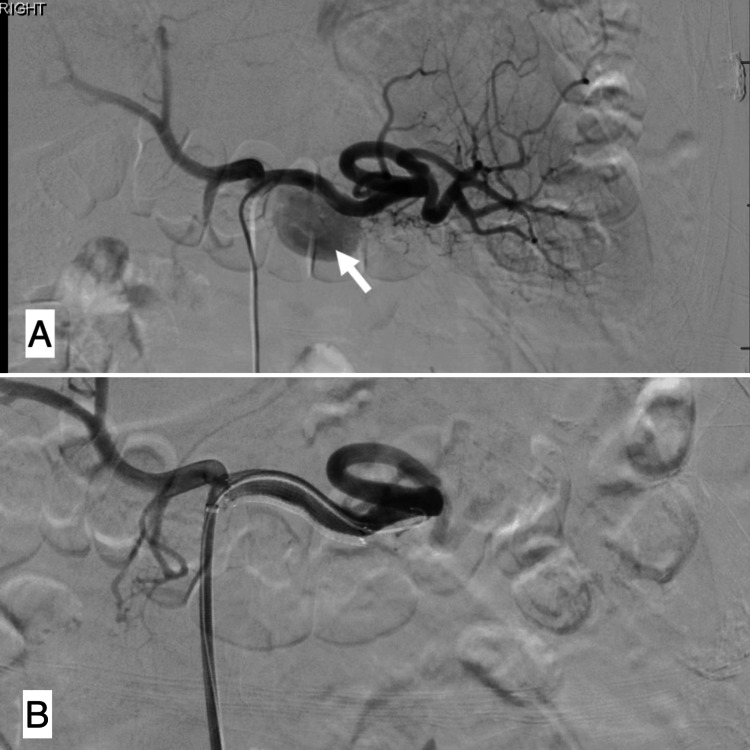
A- Fluoroscopy showing splenic artery pseudoaneurysm prior to fully-covered endovascular stent placement. B - Fluoroscopy showing splenic artery pseudoaneurysm after fully-covered endovascular stent placement

EUS revealed a 48X30 mm fluid collection around the pancreatic body that was initially suspected to be a pancreatic pseudocyst (PP) as seen in Figure 4. A pancreatic biopsy showed no evidence of autoimmune pancreatitis and normal parenchyma. It was recommended that the patient undergo an abdominal CT scan with contrast to further investigate the suspected PP. Approximately 2 weeks later before the patient could receive his recommended CT scan, the patient presented back to the ED with emesis and severe epigastric pain that radiated to his back. An immediate CT scan with contrast revealed a ruptured SAP measuring 3.6 cm with a large retro-gastric hematoma 2 cm from its origin as seen in Figure 5. The patient was immediately transferred to the closest facility with an interventional radiologist where an emergency arteriogram was conducted and a fully covered endovascular stent was successfully placed at the origin of the SAP as seen in Figure 6. During subsequent follow-up visits, lab results indicated normal lipase levels and resolved pancreatitis. Over the following 12 months, the patient denied any further epigastric pain or episodes of AP.

## Discussion

While SAPs are rare entities by themselves, the spontaneous resolution of the patient’s episodes of AP after the stenting of his SAP is of particular note as no definitive cause for his AP was determined. Patient history and appropriate diagnostic procedures ruled out all common causes of AP [6]. While hereditary and familial pancreatitis are rare etiologies of recurrent pancreatitis, the lack of any family history of pancreatitis coupled with the spontaneous resolution of the patient’s AP after his SAP treatment would not be expected if this was the etiology. Given that 18 months have passed since his SAP treatment with no further AP, it is unlikely his AP was due to a genetic issue. 

Prior cases of SAPs demonstrate AP to be the most frequent underlying cause of an SAP [6]. This traditional etiology positions SAPs as a downstream process of an AP. While this is the most likely pathophysiology in most cases, we posit that this case demonstrates that this sequence of events is reversible in which an SAP can act as an upstream process of an AP, providing a rationale for the patient's spontaneous resolution of his recurrent AP after SAP stenting. When the patient first reported to the ED, we suspect that his SAP was most likely present and was the cause of his initial episode of AP. Although mild, this initial episode of AP was significant enough to cause PVT. Over the next 6 months, his SAP expanded corresponding with his worsening episodes of AP leading up to the rupture of his SAP. 

A limitation of this case lies with the cause of the initial insulting factor for the patient's SAP. Undisclosed or unrecognized abdominal trauma could be one factor [9]. We also acknowledge the possibility of an unrecognized insulting factor that could have caused an isolated episode of AP in the patient that allowed for the development of his SAP [6]. Once this SAP developed, however, we believe this started the cycle of symptoms experienced by the patient in which the proximal location of the SAP to the pancreas and its associated inflammatory response caused compression of the pancreas and intermittent episodes of AP. Prior cases of compressive events from primary causes such as diverticula have also been shown to result in secondary AP [10,11]. If the etiology of this patient’s SAP was due to an upstream AP, the resolution of his recurrent AP would not have been expected after SAP stenting and should have persisted. We believe this case represents the first of its kind to be reported in the literature.

Upon further reflection of the patient’s EUS outlined in Figure 1, we believe that the 48X30 mm fluid collection that was suspicious of a PP was most likely an SAP. The challenging task of distinguishing between an SAP and a PP has been noted in previous cases [12]. Additionally, the SAP measured 3.6 cm on the CT scan which is approximately the same size as the fluid collection seen on EUS. This is further supported by the absence of evidence of PP on the final CT scan.

## Conclusions

The lack of any apparent etiology for the patient’s AP along with the timing of its spontaneous resolution after SAP treatment further supports this proposed etiology. This case illustrates SAP as a rare but potential cause of AP, especially in cases of unknown etiology. In future cases of recurrent episodes of idiopathic AP, an undetected SAP should be investigated as a potential etiology of AP rather than only being considered as a secondary complication.
